# Malnutrition screening on hospital admission: impact of overweight and obesity on comparative performance of MUST and PG-SGA SF

**DOI:** 10.1038/s41430-020-00848-4

**Published:** 2021-02-15

**Authors:** Iris M. Y. van Vliet, Antonio W. Gomes-Neto, Margriet F. C. de Jong, Stephan J. L. Bakker, Harriët Jager-Wittenaar, Gerjan J. Navis

**Affiliations:** 1 Department of Dietetics, University of Groningen, University Medical Center Groningen, Groningen, The Netherlands; 2 Department of Internal Medicine, Division of Nephrology, University of Groningen, University Medical Center Groningen, Groningen, The Netherlands; 3 Department of Oral and Maxillofacial Surgery, University of Groningen, University Medical Center Groningen, Groningen, The Netherlands; 4 Research Group Healthy Ageing, Allied Health Care and Nursing, Hanze University of Applied Sciences, Groningen, The Netherlands

**Keywords:** Obesity, Nutrition, Malnutrition, Risk factors

## Abstract

**Background/objectives:**

Traditional malnutrition screening instruments, including the Malnutrition Universal Screening Tool (MUST), strongly rely on low body mass index (BMI) and weight loss. In overweight/obese patients, this may result in underdetection of malnutrition risk. Alternative instruments, like the Patient-Generated Subjective Global Assessment Short Form (PG-SGA SF), include characteristics and risk factors irrespective of BMI. Therefore, we aimed to compare performance of MUST and PG-SGA SF in malnutrition risk evaluation in overweight/obese hospitalized patients.

**Subjects/methods:**

We assessed malnutrition risk using MUST (≥1 = increased risk) and PG-SGA SF (≥4 = increased risk) in adult patients at hospital admission in a university hospital. We compared results for patients with BMI < 25 kg/m^2^ vs. BMI ≥ 25 kg/m^2^.

**Results:**

Of 430 patients analyzed (58 ± 16 years, 53% male, BMI 26.9 ± 5.5 kg/m^2^), 35% were overweight and 25% obese. Malnutrition risk was present in 16% according to MUST and 42% according to PG-SGA SF. In patients with BMI < 25 kg/m^2^, MUST identified 31% as at risk vs. 52% by PG-SGA SF. In patients with BMI ≥ 25 kg/m^2^, MUST identified 5% as at risk vs. 36% by PG-SGA SF. Agreement between MUST and PG-SGA SF was low (*к* = 0.143). Of the overweight/obese patients at risk according to PG-SGA SF, 83/92 (90%) were categorized as low risk by MUST.

**Conclusions:**

More than one-third of overweight/obese patients is at risk for malnutrition at hospital admission according to PG-SGA SF. Most of them are not identified by MUST. Awareness of BMI-dependency of malnutrition screening instruments and potential underestimation of malnutrition risk in overweight/obese patients by using these instruments is warranted.

## Introduction

The impact of disease-related malnutrition on both patient outcomes and health care systems, e.g., in terms of care burden, hospital costs and length of stay, has gained increasing attention over the last decades [1–4]. In recognition of this worldwide issue, terminology and criteria for malnutrition have been defined and adjusted [5, 6], and various screening and assessment instruments have been developed and implemented to facilitate early detection and intervention [7, 8].

Although various tools are predictive of length of hospital stay, hospitalization costs and mortality, they use different criteria for risk assessment and therefore identify different patients [7–12]. Most traditional malnutrition screening instruments, including the Malnutrition Universal Screening Tool (MUST), rely considerably on criteria regarding low body mass index (BMI) and critical weight loss to identify patients in need of nutritional interventions. However, the development and implementation of these tools have been paralleled by an increasing prevalence of overweight and obesity worldwide [13]. In an increasingly overweight and obese patient population, a high contribution of criteria that depend on BMI and weight loss for detection of malnutrition may result in underdetection of disease-related malnutrition and risk thereof [14].

Previous studies suggest that malnutrition or risk thereof may be easily overlooked, especially in overweight and obese patients, and that disease-related malnutrition may be predictive of worse patient outcomes irrespective of BMI [15–17]. Moreover, clustering and interplay of risk factors can result in a cumulative risk of adverse outcomes, i.e., the co-existence of both morbid obesity and malnutrition (‘a double burden’) was found to be associated with a higher risk for developing pressure injuries [18].

Obviously, there is a need for suitable instruments to identify patients at high risk for disease-related malnutrition, presenting with a different phenotype than low BMI or critical weight loss. Such an alternative is offered by the Patient-Generated Subjective Global Assessment Short Form (PG-SGA SF) [19]. This tool can be considered as an alternative screening tool for clinical practice, as it includes items on weight history irrespective of BMI, food intake, nutrition impact symptoms (e.g., nausea, problems swallowing, diarrhea), and activity and functioning. The PG-SGA SF is part of the Full PG-SGA, which is well-implemented as a reference tool in the oncology setting and is increasingly used in hospitals worldwide [19, 20]. The PG-SGA SF has been validated as a separate tool for malnutrition screening in cancer in- and outpatients, patients with chronic kidney disease and patients prior to vascular surgery, against accepted nutritional assessment instruments such as the Subjective Global Assessment (SGA) or in association with patient outcomes such as post-operative complications [21–27]. To our knowledge, the potential role of the PG-SGA SF in malnutrition screening in a mixed hospital population, with a specific focus on its performance in overweight and obese patients, has not been explored previously.

In the current study, we aimed to assess malnutrition risk in overweight and obese hospitalized patients using both the currently used MUST and the PG-SGA SF, and to compare the performance of both instruments for malnutrition screening on hospital admission across the BMI range.

## Materials and methods

### Study population

An observational cohort study was conducted among adult patients (age ≥ 18 years) admitted to four wards of a single university hospital, i.e., the University Medical Center Groningen (UMCG), in the period of March 2016 to July 2017, as described previously in other papers based on the same cohort [28–30]. Two surgical and two medical wards were selected to reflect a mixed university hospital population. These wards had the following admitting specialisms: Otorhinolaryngology, Maxillofacial Surgery, Ophthalmology, Orthopedic and Plastic Surgery, General Internal Medicine (including Geriatric Medicine), Nephrology and Renal Transplantation, and Dermatology. Patients were excluded if they were admitted for a specialism other than those mentioned, if patients were in isolation other than contact isolation, if patients were not able to answer questions or follow instructions in Dutch or English language, or if data collection was not possible or desirable as assessed by the ward’s head nurse on duty (e.g., terminal care, severe delirium), or if data collection was completed at a previous admission.

### Data collection

Malnutrition screening using the MUST [31] was part of standard protocolized routine hospital care, as recommended by the Dutch national malnutrition guideline [32], and was performed by a nurse or food assistant within one day of admission. The MUST was originally developed for malnutrition screening in the outpatient setting, but has since been adopted by hospitals worldwide, supported by previous research on its validity in the hospital setting [11, 33]. MUST scores were extracted from the hospital database. MUST score ranges from 0 to 6 points, and malnutrition risk according to MUST was defined by MUST score categories (0 = low risk, 1 = medium risk, ≥2 = high risk), with increased risk for malnutrition defined as MUST score ≥1. MUST data were inspected for discrepancies between BMI and MUST score (i.e., BMI < 18.5 kg/m2 and MUST score 0 or 1) and accordingly, MUST score was corrected based on the patients’ height and weight in the hospital database (to MUST score ≥2 according to MUST scoring criteria).

The PG-SGA SF [34, 35] was also administered within one day of admission to assess malnutrition risk. As the PG-SGA SF was designed and deemed feasible for patients’ self-report [27, 36, 37], it was completed by the patient as much as possible. If needed, assistance was provided by a nurse, researcher, student researcher, or patient family member. The PG-SGA SF consists of four items (boxes) on weight history (Box 1, score range 0 to 5 points), food intake (Box 2, score range 0 to 4 points), nutrition impact symptoms (Box 3, score range 0 to 24 points) and activity and functioning (Box 4, score range 0 to 3 points), adding up to a total PG-SGA SF score ranging from 0 to 36 points. Malnutrition risk according to PG-SGA SF was defined by PG-SGA SF score category (0–3 = low risk, 4–8 = medium risk, ≥9 = high risk) [24], with increased risk for malnutrition defined as PG-SGA SF score ≥4.

Data regarding demographic characteristics, length of stay, and the patients’ height and weight were extracted from the hospital database. BMI in kg/m^2^ was calculated using extracted height and weight. BMI groups were defined by the WHO classification (low BMI < 18.5 kg/m^2^, normal BMI 18.5–24.9 kg/m^2^, overweight BMI 25.0–29.9 kg/m^2^, obese BMI ≥ 30.0 kg/m^2^) [38] and were dichotomized into a low/normal BMI group (BMI < 25.0 kg/m^2^) and overweight/obese BMI group (BMI ≥ 25.0 kg/m^2^) for further analyzes.

This study was performed in accordance with the Declaration of Helsinki and was approved by the Medical Ethical Committee of the UMCG (METc UMCG 2016/106). Care as usual was provided during the study period, including dietetic treatment conform hospital protocol following national guidelines [32]. In addition, an overview of PG-SGA scores was provided weekly to the ward’s head nurse or dietitian, hereby enabling intervention for patients at increased risk for malnutrition that were not yet identified by regular care protocols.

### Statistical analyses

Statistical analyses were performed using IBM SPSS Statistics for Windows, Version 23.0. Data are presented as means with standard deviations for continuous variables, as medians with interquartile range (IQR) for non-normally distributed data and ordinal variables, and as numbers with percentages for categorical data. Differences in patients characteristics and malnutrition risk between BMI groups were tested for statistical significance using ANOVA with posthoc Bonferroni tests and independent samples *t*-tests for continuous variables (age, height, weight, and BMI), chi-square tests for categorical variables (gender, ward type, MUST score category, and PG-SGA Category), and Kruskall–Wallis and Mann–Whitney *U* tests for ordinal variables (length of stay, PG-SGA score). Cohen’s kappa was used to measure the level of agreement between malnutrition risk assessment by MUST and PG-SGA SF, both overall and dichotomized for BMI group, using the interpretation previously described by Landis and Koch (<0.00 = no agreement, 0.00–0.20 = slight agreement, 0.21–0.40 = fair agreement, 0.41–0.60 = moderate agreement, 0.61–0.80 = substantial agreement and 0.81–1.00 = almost perfect agreement) [39]. Statistical significance level was set at <0.05.

## Results

### Patient characteristics

Data of 965 individual patients were collected during the study period. MUST score was available in 679 patients, and PG-SGA SF in 723 patients. Complete data on both MUST, PG-SGA SF and patient characteristics on admission were available in 430 patients, as shown in Table 1. Inconsistencies between hospital database-derived BMI and MUST score were identified in three cases (3/430 = 0.7%) and were corrected accordingly. Three percent of patients had a low BMI (<18.5 kg/m^2^) and 37% had a BMI within the normal range (18.5–24.9 kg/m^2^), 35% of the patients were overweight (BMI 25.0–29.9 kg/m^2^) and 25% were obese (BMI ≥ 30.0 kg/m^2^).

**Table 1:** Patient characteristics and risk for malnutrition for the total population and per BMI group.

Characteristics	Total *N* = 430	Low BMI <18.5 kg/m^2^ *N* = 11	Normal BMI 18.5–24.9 kg/m^2^ *N* = 161	Overweight BMI 25.0–29.9 kg/m^2^ *N* = 150	Obese BMI ≥30.0 kg/m^2^ *N* = 108	*p* value
Age (years), mean ± SD	58.4 ± 16.2	47.3 ± 15.3	55.4 ± 18.9	59.5 ± 14.7	62.3 ± 12.6	0.001*
Gender (*n*,%)						0.001*
Male	228 (53.0)	1 (9.1)	83 (51.6)	94 (62.7)	50 (46.3)	
Female	202 (47.0)	10 (90.9)	78 (48.4)	56 (37.3)	58 (53.7)	
Height (cm), mean ± SD	173.7 ± 9.4	170.2 ± 7.1	173.9 ± 9.7	174.7 ± 8.9	172.4 ± 9.7	0.151
Weight (kg), mean ± SD	81.4 ± 18.3	50.8 ± 5.7	67.6 ± 8.8	83.3 ± 8.8	102.4 ± 17.0	<0.001*
BMI (kg/m^2^), mean ± SD	26.9 ± 5.5	17.5 ± 0.9	22.3 ± 1.6	27.3 ± 1.4	34.3 ± 4.0	<0.001*
Length of stay (days), median [IQR]	5 [2–8]	5 [0–11]	5 [2–9]	5 [2–8]	6 [2–9]	0.639
Ward type (*n*,%)						0.419
Surgical	241 (56.0)	6 (54.5)	82 (50.9)	89 (59.3)	64 (59.3)	
Medical	189 (44.0)	5 (45.5)	79 (49.1)	61 (40.7)	44 (40.7)	
MUST score (*n*,%)						<0.001*
0	363 (84.4)	0 (0.0)	119 (73.9)	142 (94.7)	102 (94.4)	
1	35 (8.1)	0 (0.0)	28 (17.4)	4 (2.7)	2 (1.9)	
≥2	32 (7.4)	11 (100.0)	14 (8.7)	4 (2.7)	4 (3.7)	
PG-SGA SF score (*n*,%)						0.001*
0–3	248 (57.7)	1 (9.1)	81 (50.3)	103 (68.7)	63 (58.3)	
4–8	102 (23.7)	6 (54.5)	44 (27.3)	28 (18.7)	24 (22.2)	
≥9	80 (18.6)	4 (36.4)	36 (22.4)	19 (12.7)	21 (19.4)	
PG-SGA SF score, median [IQR]	3 [1–7]	7 [6–11]	4 [1–8]	2 [0–5]	3 [1–7]	<0.001*

^*^Significant *p* value at 0.05 level

Age was significantly higher with increasing BMI (*p* = 0.001), and there was a significant overrepresentation of women in the underweight group (*p* = 0.005). Height was similar across BMI groups, while weight and BMI differed significantly (*p* < 0.001 for all groups). There was no significant difference in distribution of patients across type of ward. Moreover, length of hospital stay was not significantly different between BMI groups.

### Prevalence of risk for malnutrition

According to MUST, 16% of all patients had increased risk for malnutrition. Distribution of risk of malnutrition assessed by MUST differed significantly between BMI groups (*p* < 0.001). According to MUST, all patients in the low BMI group (100%) had increased risk for malnutrition, which is inherent to how BMI is scored in the MUST. Prevalence of increased malnutrition risk assessed by MUST was much lower in the normal BMI group (26%), and very low in the overweight and obese BMI groups (5% and 6%, respectively).

**Fig. 1:**
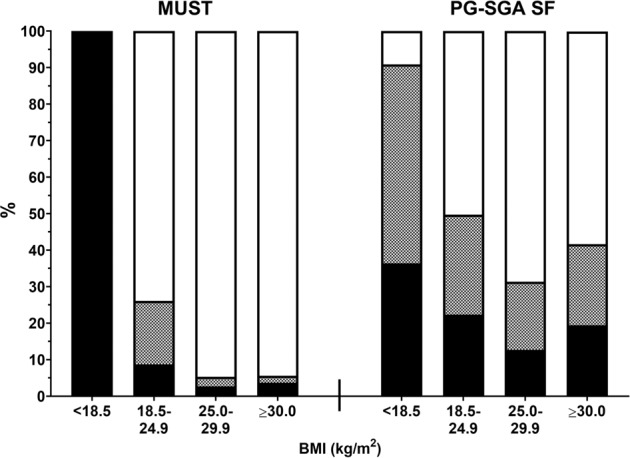
Distribution of risk for malnutrition by MUST (left panel) and PG-SGA SF (right panel) across BMI groups. Percentages of patients at high risk (black), medium risk (black-white hatched) and low risk (white) per BMI group are shown (*N* = 430).

According to PG-SGA SF, 42% of all patients had increased risk for malnutrition. Distribution of risk of malnutrition assessed by PG-SGA also differed significantly between BMI groups (*p* = 0.001). According to PG-SGA SF, almost all patients in the low BMI group (91%) had increased risk of malnutrition. Prevalence of increased malnutrition risk assessed by PG-SGA SF was lower in the normal BMI group (50%), and in the overweight and obese BMI groups (31% and 42%, respectively). Distribution of MUST score categories and PG-SGA SF score categories across BMI groups are visualized in Fig. 1.

### MUST vs. PG-SGA SF

In the comparison of MUST score category vs. PG-SGA SF score category, slight overall agreement between instruments (*к* = 0.143, *p* < 0.001) was found. When stratifying data BMI into two groups (low or normal BMI as a group and overweight or obese BMI as a group), the slight agreement for the comparison of MUST score category vs. PG-SGA SF score category remained for both groups (*к* = 0.171, *p* = 0.001 and *к* = 0.056, *p* = 0.049 respectively). Conversely, 90% (83/92) of patients with increased risk for malnutrition according to the PG-SGA SF, had low risk according to the MUST.

**Table 2:** **a** Risk for malnutrition by MUST score category vs. PG-SGA SF score category in patients with low or normal BMI (*N* = 172). **b** Risk for malnutrition by MUST score category vs. PG-SGA SF score category in patients with overweight or obesity (*N* = 258).

		MUST score category			
		0 Low risk	1 Medium risk	≥2 High risk	Total
**BMI** **<** **25.0** **kg/m**^**2**^					
PG-SGA SF category	0–3 *Low risk*	68	12	2	82
	4–8 *Medium risk*	33	7	10	50
	≥9 *High risk*	18	9	13	40
	Total	119	28	25	172
**BMI** **≥** **25.0** **kg/m**^**2**^					
PG-SGA SF category	0–3 *Low risk*	161	3	2	166
	4–8 *Medium risk*	50	0	2	52
	≥9 *High risk*	33	3	4	40
	Total	244	6	8	258

### Identification of malnutrition risk in overweight or obese patients using the PG-SGA SF

Of the 258 patients with a BMI in the overweight or obese range, 36% had increased risk for malnutrition at hospital admission according to the PG-SGA SF. Overweight or obese patients with increased risk for malnutrition were significantly more often female (*p* = 0.007), had significantly lower body height (*p* = 0.001), had a significantly higher median hospital length of stay (*p* = 0.002), and were significantly more often admitted to a medical ward (*p* < 0.001) than overweight or obese patients with low risk for malnutrition. Patients with increased risk for malnutrition also were slightly older than low risk patients, but this difference was not statistically significant (*p* = 0.082). Body weight, BMI, and BMI classification were also not significantly different between overweight or obese patients with low or increased risk for malnutrition. Inherently, overweight or obese patients with increased risk for malnutrition had a significantly higher PG-SGA SF score compared to patients with low risk (median score 8 vs. 1, *p* < 0.001). The scores on all components (boxes 1–4) of the PG-SGA SF were also significantly different between patients at low or increased risk for malnutrition (*p* < 0.001 for all components). The largest difference was found in Box 3 Symptoms (median score 5 vs. 0), followed by Box 4 Activities and functioning (median score 2 vs. 0) and Box 2 Intake (median score 1 vs. 0).

## Discussion

In this mixed hospital population, MUST identified 16% and PG-SGA SF identified 42% as having increased risk for malnutrition at hospital admission. In patients with overweight or obesity, these figures were 5% and 36%, respectively. Of 92 overweight or obese patients at risk for malnutrition according to the PG-SGA SF, a striking 90% was categorized as low risk by the MUST. Female overweight or obese patients, and patients admitted to a medical ward were more often at risk, and length of hospital stay was significantly higher in overweight or obese patients at increased malnutrition risk compared with patients with low malnutrition risk and a similar BMI. Difference in identification of high risk patients was mostly due to assessment of nutrition impact symptoms and activity and function in the PG-SGA SF.

In our study, prevalence of malnutrition risk among overweight or obese hospitalized patients was somewhat higher (36% by PG-SGA SF) compared with previous studies performed in Australia and New Zealand (31% by MST; 18% malnourished by SGA) [16] and Israel (24% by NRS 2002) [17]. Prevalence of overweight or obesity was also somewhat higher in our sample compared to the previous studies, which may reflect a geographical difference, a time trend (the current data was collected in 2016/2017 vs. 2010 in previous studies), or perhaps both. Moreover and most importantly, different instruments were used to assess malnutrition risk. The use of different instruments also likely has resulted in a difference in prevalence, since the PG-SGA SF, MST, SGA, and NRS 2002 use different criteria and cutoffs for risk assessment.

In the PG-SGA SF, presence of nutrition impact symptoms largely contributes to the total risk score, in contrast to for example MST, MUST, or NRS 2002. In the current study, we found that overweight and obese patients at increased risk for malnutrition scored a median of 5 points on presence of nutrition impact symptoms, compared to 0 points in the patients with low risk for malnutrition. In patients with increased risk, this score indicates presence of two to five different nutrition impact symptoms, e.g., nausea, problems swallowing, diarrhea [34, 35]. Monitoring of nutrition impact symptoms may aid in proactive and early detection and intervention, to prevent deterioration of nutritional status and future malnutrition. For example, previous research has shown that nutrition impact symptoms are prevalent in patients with cancer [40], and symptoms presented before treatment negatively affect intake, weight and functional capacity [41]. In patients with chronic liver disease, nutrition impact symptoms are also prevalent, and have been associated with malnutrition and worse quality of life [42].

Different nutrition experts have previously advised to take caution with regard to BMI as a measure for nutritional status at the individual level, as BMI does not differentiate between body weight components. Assumptions on the stability of body weight components, specifically muscle mass and fluid status, are often not met in patient populations, and underlying muscle mass depletion may be masked by excess fat mass and/or fluid accumulation [43]. Relying solely on BMI for risk assessment may therefore lead to underdetection of disease-related malnutrition [44], as previously shown in, e.g., patients on hemodialysis [45], COPD [46], or liver cirrhosis [47]. Therefore, ideally malnutrition risk screening is followed by a nutrition assessment, including body composition assessment, to identify malnourished patients presenting with various phenotypes, as is also recommended by the GLIM [6].

Previous studies have compared malnutrition screening outcomes to body composition assessment using gold standard methods for determining muscle mass, and underscore the importance of assessment in addition to malnutrition screening alone. In a study of 363 patients undergoing surgery for colorectal cancer, prevalence of increased malnutrition risk was 21% according to the MUST, while low skeletal muscle index (SMI) was present in 50% of patients as determined by CT. While there was a significant association between increased risk of malnutrition and presence of low SMI, 45% of the patients with low risk of malnutrition had a low SMI [48]. Similar results were reported in patients with solid tumors and patients with rheumatoid arthritis, indicating poor performance of the MUST in detecting low muscle mass [49, 50]. Previous studies suggest a somewhat higher concordance between increased risk of malnutrition according to the PG-SGA (SF) and low muscle mass as determined by a gold standard assessment method, although on an individual level there is still a considerable risk of misclassification. In a study of 1157 overweight or obese cancer patients, prevalence of increased risk of malnutrition was 64% according to the PG-SGA SF, while low SMI was present in 42% of patients as determined by CT. While there was significant overlap between increased risk of malnutrition and low SMI, 36% of the patients with low risk of malnutrition had low SMI [51].

In the current study, we compared the performance of two previously validated screening tools across the range of BMI without using a gold standard method for the diagnosis of malnutrition and/or reduced muscle mass, which can be regarded as a limitation of our study. Although imaging methods, i.e., CT and MRI, are considered the gold standard techniques to assess muscle mass [6, 52], there are several limitations to their use for body composition assessment in clinical practice. These include high costs, practical considerations such as not being able to move the equipment, and, for CT, radiation exposure [53, 54]. Alternative recommended techniques such as DXA are also not portable, and while bio-electrical impedance analysis is suitable for bedside use, it is considered to lack validity in patients with obesity [54, 55]. Therefore, other methods for bedside measurement, such as ultrasound, are gaining interest, but require further investigation [56]. Moreover, 24-h urine collections allow assessment of muscle mass from excretion of creatinine, but are only available in specific settings [57–59]. Thus, although additional body composition assessment is required to make an adequate diagnosis of malnutrition, its application in daily practice remains challenging.

Our findings should be interpreted with caution due to a lack of comparison with a “gold standard” comparator for the diagnosis of malnutrition, but point towards a multidimensional approach, in which other risk factors or facets such as nutrition impact symptoms, activity and function are more prominent components. As malnutrition screening is the first step in the nutrition care pathway and determines further assessment, intervention and monitoring actions [6], the choice of screening tool is pivotal for quality of nutritional care. In our hospital and in many other care settings worldwide, the MUST has been adopted because of its ease and simplicity for clinical practice. However, our results indicate that the use of the MUST has limitations in a predominantly overweight and obese population because of its prominent BMI-criterion. Other screening tools that depend largely on BMI for risk assessment may have the same limitations, and future research is needed to further optimize malnutrition screening policy, adapting to a changing hospital population. Our results suggests that multidimensional BMI-independent screening tools such as the PG-SGA SF might therefore be more suitable compared with traditional BMI-dependent tools such as the MUST, to detect malnutrition risk in patients across the BMI range. However, for implementation of the PG-SGA SF in routine hospital care, additional studies regarding efficacy, cost-effectiveness, and feasibility on an organizational level are needed. The increasing prevalence of obesity worldwide supports the clinical relevance of such studies.

To our knowledge, the current study is the first to compare malnutrition screening by PG-SGA SF and MUST, with specific focus on detection of malnutrition risk in patients with a BMI in the overweight or obese range. The use of data from clinical practice has provided us with more insight on this matter, however, this is accompanied by a relatively large percentage of missing data, resulting in potential bias. Another limitation of the current study is the lack of a “gold standard” for the diagnosis of malnutrition to test our findings against. Therefore, we cannot infer definite conclusions on which tool is “correct”, but our findings illustrate and raise awareness for the potential consequences of using BMI-dependent screening tools in an increasingly overweight and obese patient population. However, to put our findings in perspective, we are currently analyzing the predictive value of the current data for patient outcomes such as readmission and mortality. In a previous preliminary analysis of this data, we showed that increased malnutrition risk as assessed by PG-SGA SF was significantly associated with prolonged hospitalization, readmission, and mortality within 6 months after discharge, independent of age, sex, and hospital ward. Malnutrition risk by MUST was associated with higher risk of mortality, but not with prolonged hospitalization or risk of readmission [29, 30]. These findings regarding the predictive validity of the PG-SGA SF are consistent with previous research in cancer patients, in which high risk of malnutrition by PG-SGA SF was associated with a longer length of hospital stay, a dose reduction in chemotherapy, and increased mortality [22]. Another limitation of the current study is that we did not collect more in-depth data on PG-SGA SF individual item scores and patient characteristics such as ethnicity, and therefore were not able to analyze all individual items of the PG-SGA SF or cross-check patient generated data on weight and height with hospital database data. Also, we therefore could not adjust BMI cutoffs for ethnicity. However, it is unlikely that this has largely influenced our results, since we know from experience that the majority of patients admitted to our hospital is of Caucasian ethnicity.

In conclusion, increased malnutrition risk is prevalent on hospital admission across the BMI scale, including more than one-third of patients in the overweight or obese range. Among the latter, only a small minority is identified by the currently used MUST. Alternative tools, less dependent on BMI, such as the PG-SGA SF, should be considered for initial malnutrition screening and additional nutrition assessment is recommended, to prevent malnutrition from being undetected and untreated in all patients, regardless of their BMI.
